# New Insights Into the Role of β-NGF/TrKA System in the Endometrium of Alpacas During Early Pregnancy

**DOI:** 10.3389/fvets.2020.583369

**Published:** 2021-01-22

**Authors:** Daniela E. Barraza, Luciana M. Sari, Silvana A. Apichela, Marcelo H. Ratto, Martin E. Argañaraz

**Affiliations:** ^1^Instituto Superior de Investigaciones Biológicas (INSIBIO), Consejo Nacional de Investigaciones Científicas y Técnicas - Universidad Nacional de Tucumán (CONICET-UNT), and Instituto de Biología “Dr. Francisco D. Barbieri”, Facultad de Bioquímica, Química y Farmacia, UNT, San Miguel de Tucumán, Argentina; ^2^Cátedra de Zootecnia General I, Facultad de Agronomía y Zootecnia, UNT, San Miguel de Tucumán, Argentina; ^3^Facultad de Ciencias Veterinarias, Universidad Austral de Chile, Valdivia, Chile; ^4^Cátedra de Biología Celular y Molecular, Facultad de Bioquímica, Química y Farmacia, UNT, San Miguel de Tucumán, Argentina

**Keywords:** alpacas, embryo implantation, uterus, β-NGF, VEGF

## Abstract

One striking reproductive feature in South American camelids is that more than 90% of gestations are established in the left uterine horn (LUH). This phenomenon could be related to a differential vascular irrigation of the LUH. An increase of vascularization in llama endometrium was observed after systemic administration of Beta Nerve Growth Factor (β-NGF), a neurotrophin present in the uterus and placenta of various mammals that is involved in pregnancy development. We hypothesized that the β-NGF signaling pathway is related to embryo implantation in the LUH in camelids. The aim of this study was to characterize the spatial expression of β-NGF and its high-affinity receptor, TrKA, between LUH and right uterine horn (RUH) of non-pregnant (NP) and early pregnant alpacas (15 and 30 days of gestation, 15 and 30P, respectively). In addition, β-NGF, TrKA, and Vascular Endothelium Growth Factor A (VEGFA) temporal gene expression patterns and counting of blood vessels were evaluated among groups. The β-NGF and TrKA were localized in the luminal, glandular, and vascular epithelium of the alpaca uterus and in the embryonic membranes of the 30-days-old conceptus. β-NGF and TrKA immunosignal were stronger in 15P females than that of NP and 30P. In addition, TrKA signal was higher in the LUH luminal epithelium of NP and 15P alpacas than that of NP-RUH and 15P-RUH. β-NGF mRNA relative abundance was higher in the 30P-RUH than that of NP-RUH; whereas TrKA mRNA abundance only differed between 15P-RUH and NP-LUH. VEGFA mRNA relative abundance was higher in NP females compared to the LUH of 15P and 30P alpacas, and lower to their right counterparts. The number of vessels per field was higher in 15P than that of 30P. A positive correlation was observed between the number of vessels per field and β-NGF immunosignal in 15P-LUH. In contrast, the area occupied by vessels was higher in 30P alpacas than of NP and 15P females. The changes of β-NGF/TrKA expression pattern in the peri-implantation endometria between LUH and RUH and their localization in the extraembryonic membranes support the implication of the neurotrophin during implantation and pregnancy development in South American Camelids.

## Introduction

In the reproductive physiology of mammals, critical events related to embryo survival occur during early pregnancy, with the majority of embryo losses occurring in the first month of pregnancy (1, 2). In South American camelids (SACs), the pregnancy rate 30 days after mating is <50% (3), indicating that embryo losses are much higher in SACs than in other small ruminants (4). Particularly, in SACs, the embryos formed in the right oviduct must first migrate to the left uterine horn (LUH) to subsequently achieve their successful implantation. In alpacas, embryos reach the uterus on 6 days post ovulation (dpo) and by 9 dpo, 83% of embryos derived from right-ovary ovulations have arrived to the LUH. Maternal recognition of pregnancy occurs between 8 and 10 dpo, conceptus elongation between 10 and 15 dpo, and the apposition and implantation start around 20 dpo (5–7). If an embryo implants in the right uterine horn (RUH) gestation probably would not continue, since RUH would seem to be inadequate to sustain pregnancy (8, 9), (since 98% are carried out in LUH, a 2% do actually get to term in RUH) (10, 11). It has been proposed that this unique pattern of early embryo migration and implantation is influenced by a vascular asymmetry favoring the blood vessels that irrigate the LUH (12). Interestingly, Urra et al. (13) reported an increase in endometrial vascularization in the llama uterus, after the intrauterine administration of beta nerve growth factor (β-NGF).

The β-NGF, a member of the neurotrophin family, would seem to be a key factor for the success of pregnancy since it constitutes a functional link between the nervous, immune, and endocrine systems (14). The multiple effects of β-NGF are mediated through its receptors, the high-affinity membrane receptor tyrosine kinase A (TrkA) and the low-affinity receptor, the nerve growth factor receptor (NGFR or p75NTR) (15–17). The expression of β-NGF and its receptors in a wide variety of reproductive tissues, including different organs of the male and female genital tract, enables it to perform key reproductive functions.

This system is present in the uterus of several species such as rat (18), squirrel (15), ewe (19), horse, pig, human (20), and induced ovulatory species such as rabbits and camelids (17). Several reports suggest that this factor would participate in the endometrial prostaglandin synthesis during the maternal recognition of pregnancy, early development of the embryo, and conceptus immunotolerance (21, 22). Furthermore, in humans and rats, β-NGF has been shown to be a potent angiogenic factor, increasing the expression of vascular endothelial growth factor (VEGF) and promoting vascular cell proliferation in female reproductive organs (23, 24). The VEGFA, a member of the VEGF family, is a marker of uterine receptivity for implantation in humans and bovines. It mediates the establishment of an appropriate uterine environment through the regulation of endothelial cell growth, angiogenesis, and vascular permeability (25).

In camelids, β-NGF is present in the seminal plasma acting as an ovulation-inducing factor (26, 27). The factor is absorbed from the endometrium surface after copulation, rapidly entering systemic circulation to elicit GnRH secretion from the hypothalamus (28). Moreover, the factor increases uterus vessel formation and *corpus luteum* vascularization. In fact, β-NGF addition to llama cultured granulosa cells increased the expression of *VEGFA* after 48 h *in vitro* culture (29).

The mechanisms leading to embryo implantation and survival in SACs is far from being explained. Given this scenario, associated with the reports on the roles of β-NGF in the uterus during pregnancy, the objectives of this study were: (1) Characterize the spatial localization and expression pattern of β-NGF and TrkA in the endometrium of LUH and RUH in alpacas during the first month of gestation. (2) Describe the VEGFA gene expression pattern and count the number of associated blood vessels, and its association with β-NGF expression.

## Materials and Methods

### Animals and Sampling

Twelve adult female Huacaya breed alpacas (*Vicugna pacos*), >2 years of age, weighing 65–70 kg and that were destined for meat production, were used in this study. According to their reproductive status, females were divided into 3 experimental groups: non-pregnant (NP; *n* = 4), 15 days of pregnancy (15P; *n* = 4), and 1 month of pregnancy (30P; *n* = 4).

Non-pregnant and 15 days-pregnant alpacas were obtained from the Veterinary Research Center (IVITA), Universidad Nacional Mayor de San Marcos (UNMSM) in the province of Canchis, Perú (14°S, 71°W; 3698 m altitude). Virgin females, between 2 and 3 years of age, were mated once with a fertile adult male and they were slaughtered fifteen days after mating, in line with standards set by the local Committee of Animal Ethics and Welfare (Comité de Ética y Bienestar Animal; CEBA from the School of Veterinary Medicine of the UNMSM). Females with follicles of <7 mm in diameter in their ovaries were considered NP; while, those with a *corpus luteum* and embryos in the uterus were considered pregnant (Table 1).

**Table 1:** Reproductive features of alpaca's experimental groups.

**Physiological status**	***N***	**Follicle/CL diameter (mm)**	**Left side follicle/CL (%)**	**Embryo (mm)**	**Left side pregnancy (%)**
NP	4	8.5 ± 1.8	75	NA	NA
15P	4	14.5 ± 1.3	50	18.5 ± 1.1^**^	NA
30P	4	16.0 ± 1.0	50	16.5 ± 1.5^*^	100

*NP, non-pregnant; 15P, 15 days-pregnant; 30P, 30 days-pregnant; CL, corpus luteum*.

*NA, not applicable*.

** *Elongated embryo*.

* *Crown-rump length*.

Alpacas around 1 month of pregnancy (30P) were obtained from a slaughterhouse at Huancavelica, Perú (12°S, 74°W, 3676 m altitude). The gestational age (GA) of these animals was calculated using embryo crown-rump length (CRL) and the formula GA= (10.328^*^CRL)^0.4427^ (Table 1) as it was previously reported (6, 7, 30, 31). Based on previous studies maternal recognition of pregnancy occurs between 8 and 10 dpo and embryo apposition happens around 20 dpo (5, 30). Therefore, 15P samples corresponded to the pre-implantation stage; while in 30P samples the embryo was already implanted in the uterus.

Endometria from the midsection of the LUH and RUH were dissected in two segments of 10 mm, which were subsequently placed in 4% formaldehyde-PBS solution (pH 7.4) for immunohistochemical assays or RNAlater® (Ambion, Austin, USA) for RT-PCR assays. RNAlater®-embedded samples were transported on dry ice to the laboratory at INSIBIO, Argentina and stored at −80°C until further analysis.

### RNA Isolation and cDNA Synthesis

Total RNA from endometria was extracted using the Spin Vacuum total RNA Isolation System™ (Promega, Madison, USA) according to the manufacturer's instructions. The RNA samples were quantified at 260 nm on a Shimadzu UV- spectrophotometer 1800 (Shimadzu Corporation, Japan) and its integrity was examined by electrophoresis on 1.5% agarose gels stained with Invitrogen™ SYBR™ Safe DNA Gel Stain (Carlsbad, USA). Finally, total RNA was stored at −80°C.

Reverse transcription of all of the RNA samples was carried out with M-MLV reverse transcriptase™ (Promega) and Oligo(dT)15-primer™ (Promega) in a 25 μl reaction mixture. Reactions were performed by incubating the mixture in a thermocycler (Techne™ TC-512 Gradient Thermal Cycler, Burlington, USA) at 42°C for 90 min followed by a reverse-transcriptase inactivation at 94°C for 5 min.

### Semi-quantitative PCR of *β-NGF, TrKA, and VEGFA*

The relative abundance of β*-NGF, TrKA*, and *VEGFA* mRNA was analyzed by semi-quantitative PCR in NP (*n* = 4), 15P (*n* = 4), and 30P (*n* = 4) endometria using *ACTB* as reference gene as was described by Barraza et al. (32).

PCR amplifications were carried out in a final volume of 10 μl containing 0.5 μL of cDNA, 2 μL of 5X Green GoTaq® Reaction Buffer (Promega), 0.2 mM of dATP, dTTP, dCTP and dGTP (Promega), 2.5 units of GoTaq® DNA polymerase (Promega), and 1 μM of each primer pair: β*-NGF (5*′*-TGCTGGGAGAGGTGAACATT-3*′*, 5*′*-CGAAGGTGTGGGTTGTGGTA-3*′*), TrKA (5*′*-GCTTCATCTTCACCGAGTTCCT-3, 5*′*-TAGCCAGCAGCGTGTAGTTG-3*′*), VEGFA (5*′*-CGGTATAAATCCTGGAGCGT-3*′*, 5*′*-GCCTCGGCTTGTCACATCT-3*′*)* and *ACTB (5*′*-GCGGGACCACCATGTACC-3*′*, 5*′*-ACTCCTGCTTGCTGATCCAC-3*′*)*. Oligonucleotides were synthetized at Thermo Fisher Scientific Custom Standard DNA Oligos Service (Buenos Aires, Argentina).

Different amplification settings were assayed to determine optimal PCR conditions: 94°C for 3 min, followed by 40 cycles at 94°C for 10 s, 60°C for 5 s, 72°C for 5 s, and a final extension at 72°C for 5 min. PCR products were analyzed with 1.5% agarose gel electrophoresis.

For semi-quantitative expression analysis, the 4 samples of each uterine horn and physiological status were amplified in duplicate (*n* = 4, *r* = 2). The amplification products were visualized in agarose gels and documented with a Pentax™Optio™ M90 digital camera (Milan, Italy), and the optical density of PCR products was quantified using ImageJ 1.42q software (National Institutes of Health, Bethesda USA). Data represent the average of the 2 PCR amplifications. The relative abundance of each transcript was normalized against that of *ACTB*, and the transcript/*ACTB* ratio was calculated.

### Immunohistochemistry

Immune assays were performed as described by Sari et al. (33). Fixed tissues were dehydrated in ethanol, cleared in chloroform, and embedded in paraffin blocks. Five-micron sections were cut and mounted on Biotraza positively charged slides (Huida Medical Instruments CO., Jiangsu, China). After deparaffinization with xylene and rehydration, sections were subjected to antigen retrieval by incubating in Proteinase K (Sigma-Aldrich, St. Louis, USA) solution (10 μg/μL in Tris EDTA (TE) Buffer, 50 mM Tris Base, 1 mM EDTA, 0.5% Triton X-100, pH 8.0) for 20 min at 37°C in a humid chamber, followed by incubation in TE Buffer at room temperature (RT) for 10 min. The slides were then blocked with 1% Bovine Serum Albumin (Sigma-Aldrich) in PBS at room temperature for 30 min. Then, they were incubated at 37°C for 1 h with polyclonal antibodies against β-NGF (dilution 1:1,500, sc-548) or TrKA (dilution 1:100, sc-118, Santa Cruz Biotechnology, Santa Cruz, USA). After washing in 0.02% Tween-PBS, slides were incubated for 20 min at RT in a humidified chamber with a 1:200 dilution of the biotinylated anti-rabbit IgG antibody (B8895, Sigma-Aldrich). Subsequently, samples were incubated at RT for 30 min with a solution 1:500 ExtraAvidin®-Alkaline Phosphatase (Sigma-Aldrich). Following three washes with 0.02% Tween in PBS, the sections were incubated with SIGMAFAST™ BCIP®/NBT (B5655, Sigma-Aldrich) until color was developed.

Finally, the sections (*n* = 4) were counterstained with Nuclear Fast Red (N3020, Sigma-Aldrich), then dehydrated and mounted with Entellan® (Merck, Darmstadt, Germany). Negative controls were performed by replacing the primary antibody with blocking buffer with 1% BSA-PBS with 0.02% Tween under the same experimental conditions.

Samples were observed under a Leica DM500^TM^ light microscope, and images were captured with a Leica ICC50 ^TM^ HD camera using LASZ Leica Inc. Software.

The ImageJ 1.42q software (National Institutes of Health, Bethesda USA) was used to measure the immunolabeled area of each sample according to Jensen (34). For this analysis, three different photographs (100X) of each section were used. Three rectangular regions of interest (ROI) were randomly selected in each image for its evaluation. First, the images were converted to 8 bits, then a specific threshold was determined and quantification was performed. Data were expressed as pixels/μm^2^.

### Histological Evaluation of Blood Vessels

The number and area occupied by vascular vessels were determined as follows: endometrial sections of 5 μm were stained with hematoxylin and eosin (BIOPUR, Rosario, Argentina). Then, images were captured with a DM500^TM^ microscope coupled to a Leica ICC50^TM^ HD camera, and the images were converted to 8 bits. Each vessel was marked in black and ImageJ v1.42q (National Institutes of Health, Bethesda USA) was used to analyze the number of marked vessels/field and the total area of vascularization (35). Nine representative microscopic fields of each uterine horn (*n* = 4) were randomly selected for all measurements. Data represent the average of the nine measurements at 100X magnification. Data were expressed as a percentage per field of endometrium.

### Statistical Analysis

Statistical analysis was performed with InfoStat software (Universidad Nacional de Córdoba, Córdoba, Argentina, see http://infostat.com.ar). Gene relative abundance as well as immunohistochemistry staining were analyzed with One-way Analysis of Variance (ANOVA) followed by Tukey *post-hoc* test, which is a multiple comparison test. The power of each performed test with α = 0.050 was between 1.0 and 0.8; 0.80 is the lowest desired power value. Less than desired power indicates there are less likely possibilities to detect a difference when one actually exists. On the other hand, the number of vessels and the vascularized area were evaluated using the Kruskal–Wallis test (a non-parametric, distribution free test). Results are expressed as the mean ± standard error (SE). In addition, correlation analysis between the variables was performed for each uterine horn, without discriminating between physiological status. Pearson Correlation Coefficient was calculated. In all the analyses, data were considered statistically significant at *p* < 0.05.

## Results

### Relative Abundance of *β-NGF* and *TrKA* mRNA in Alpaca Endometrium

The presence of β*-NGF* and *TrKA* transcripts was found in the uterine horns of alpacas in both pregnant and non-pregnant females.

**Figure 1:**
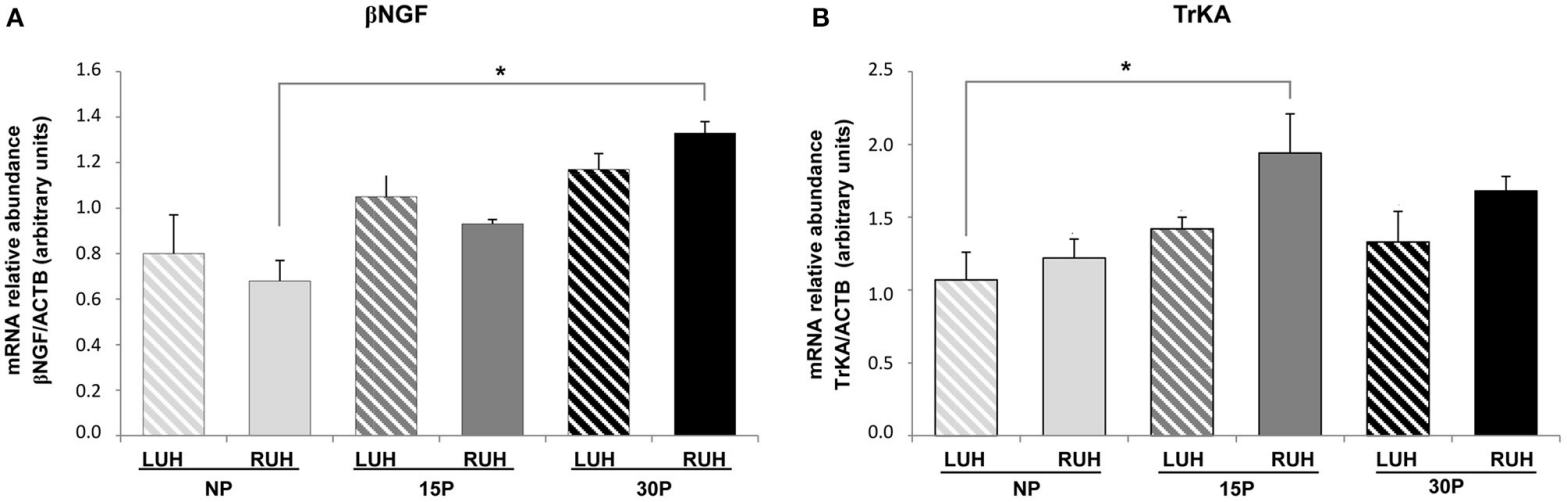
Relative abundance of β*-NGF* **(A)** and *TrKA* **(B)** transcripts in the endometrium of non-pregnant (NP), 15 days-pregnant (15P), and 30 days-pregnant (30P) alpacas by RT-PCR. Histograms illustrate mean values ± SE. Asterisks denote significant differences (*p < *0.05*). LUH (left uterine horn) and RUH (right uterine horn).

The relative abundance of β*-NGF* showed similar levels between the left and right uterine horns in all the experimental groups. When the physiological status was compared, the RUH of 30P animals displayed higher relative abundance levels than the RUH of NP females, *p* = 0.008. While β*-NGF* relative abundance in the LUH presented steady levels in all the groups (Figure 1A).

Regarding *TrKA*, transcript relative abundance was higher in the right uterine horn of 15P females compared to NP-LUH, *p* = 0.025 (Figure 1B).

### Immunolocalization of β-NGF and TrKA in Alpaca Endometrium

**Figure 2:**
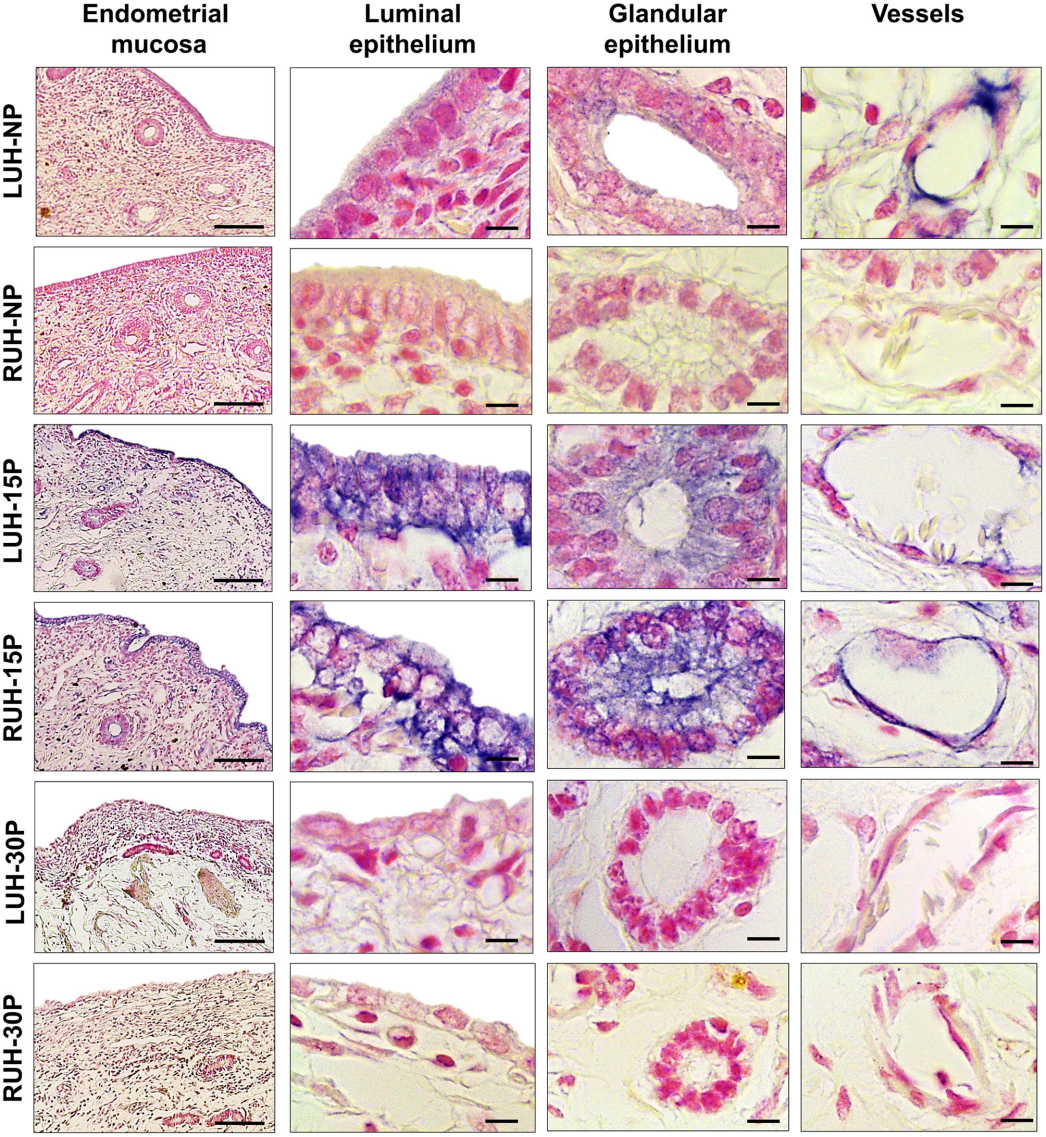
Immunohistochemical localization of β-NGF in the endometrial mucosa of alpacas, positive labeling gives a blue-violaceous coloration. A 10X magnification of endometria of the left uterine horn (LUH) and right uterine horn (RUH) of non-pregnant females (NP); 15 days-pregnant (15P); and 30 days-pregnant females (30P) is presented (scale bar = 100 μm). Also, a 100X magnification of the luminal, glandular, and vascular endothelium of the different physiological status is shown (scale bar = 10 μm).

β-NGF tissue localization and immunolabeling intensity changed during the peri-implantation period; the signal was more intense in the uterine horns of 15P alpacas compared to the uterine horns of NP and 30P females. In non-pregnant females, the β-NGF mark was faint and located in the luminal and glandular epithelium as well as the endothelium of both uterine horns. NP-LUH showed a stronger immunosignal compared to NP-RUH at the luminal epithelium. In 15 day-pregnant alpacas, an intense immunolabel was observed mainly in the luminal epithelium. A mark was also present in the glandular epithelium and the endothelium. No differences were observed between the left and right uterine horns. In 30 day-pregnant animals, β-NGF staining was weak in the endometrium, no differences between LUH and RUH were detected (Figure 2 and Table 2).

**Table 2:** Densitometry analysis of β-NGF immunolabeling in alpaca endometria.

**β-NGF**		**Luminal epithelium**	**Glandular epithelium**	**Vascular epithelium**	**Total immunolabeling**
NP	LUH	30140 ± 65.39^b,A^	29986 ± 29.78^a^	19725 ± 29.18^b^	79851 ± 97.13^b,A^
	RUH	6214 ± 9.37^a,B^	9433 ± 3.69^a^	8051 ± 14.35^a.b^	23698 ± 10.52^a,B^
15P	LUH	78435 ± 67.39^c^	81881 ± 33.12^b^	60675 ± 43.54^c^	220991 ± 104.71^c^
	RUH	74510 ± 20.12^c^	80920 ± 159.33^b^	53315 ± 62.76^c^	208745 ± 225.73^c^
30P	LUH	5109 ± 2.08^a^	3653 ± 2.57^a^	3929 ± 8.46^a^	12692 ± 8.57^a^
	RUH	4687 ± 5.26^a^	3427 ± 9.58^a^	4819.4 ± 3.54^a.b^	12934 ± 8.03^a^

*Mean values (pixels/μm^2^) ± SE are presented, comparisons are made within each column. When the physiological status are compared the significant differences (p < 0.05) are indicated by different lowercase letters (a, b, c). The statistical differences (p < 0.05) between LUH and RUH within NP (non-pregnant), 15P (15 days-pregnant) and 30P (30 days-pregnant) status are indicated by different capital letters (A, B). LUH, Left uterine horn; RUH, right uterine horn.

**Figure 3:**
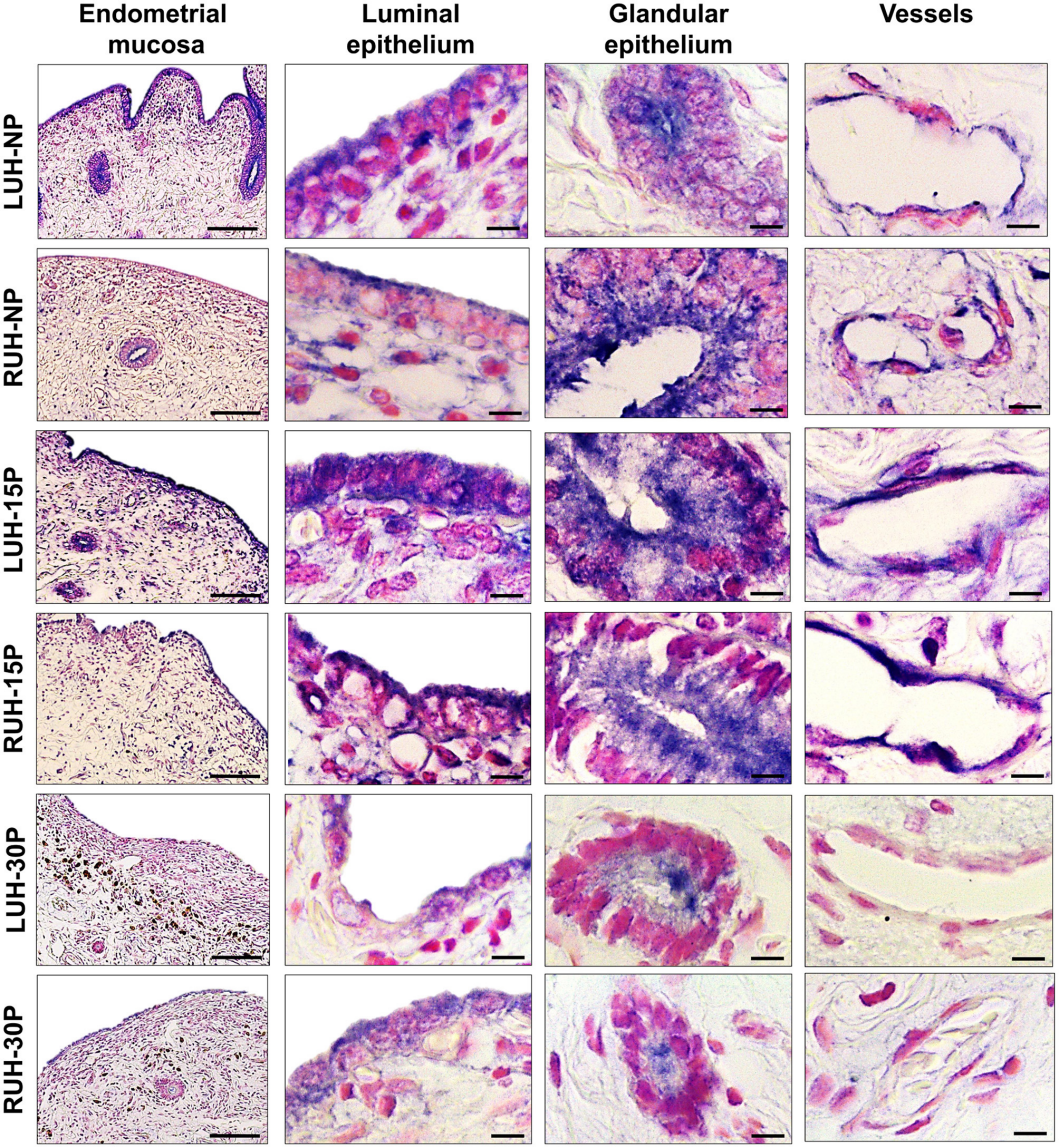
Immunohistochemical localization of TrKA in the endometrial mucosa of alpacas, positive labeling gives a blue-violaceous coloration. A 10X magnification of endometria of the left uterine horn (LUH) and right uterine horn (RUH) of non-pregnant females (NP); 15 days-pregnant (15P); and 30 days-pregnant females (30P) is presented (scale bar = 100 μm). Also, a 100X magnification of the luminal, glandular and vascular endothelium of the different physiological status is shown (scale bar = 10 μm).

TrKA mark was co-localized with β-NGF. The receptor immunolabel was detected in the luminal, glandular, and vascular epithelium. The same distribution was observed in pregnant and non-pregnant alpacas and between left and right uterine horns (Figure 3). However, the mark was stronger in the uterine horns of 15P females compared to NP and 30P animals. Statistically significant differences between LUH and RUH were observed only at the luminal epithelium of NP and 15P alpacas, where the signal was more intense in the LUH (Table 3).

**Table 3:** Densitometry analysis of TrKA immunolabeling in alpaca endometria.

**TrKA**		**Luminal epithelium**	**Glandular epithelium**	**Vascular epithelium**	**Total immunolabeling**
NP	LUH	102691 ± 58.71^b.c,A^	110213 ± 72.76^a^	47758 ± 148.23^b^	260663 ± 205.68^b.c^
	RUH	61587 ± 60.11^a,B^	89403 ± 45.13^a^	47950 ± 27.85^b^	198940 ± 90^a.b^
15P	LUH	108196 ± 78.62^c,A^	151973 ± 35.88^b^	85953 ± 26.31^c^	346123 ± 108.34^d^
	RUH	75620 ± 58.33^a,B^	147180 ± 115.85 ^b^	64857 ± 91.01^b.c^	287657 ± 258^c.d^
30P	LUH	67204 ± 24.84^a^	89689 ± 60.97 ^a^	3909 ± 3.89^a^	160803 ± 82.38^a^
	RUH	79176 ± 27.92^a.b^	101597 ± 79.04^a^	3789 ± 6.26^a^	151814 ± 68.71^a^

*Mean values (pixels/μm^2^) ± SE are presented, comparisons are made within each column. When the physiological status are compared the significant differences (p < 0.05) are indicated by different lowercase letters (a, b, c, d). The statistical differences (p < 0.05) between LUH and RUH within NP (non-pregnant), 15P (15 days-pregnant) and 30P (30 days-pregnant) status are indicated by different capital letters (A, B). LUH, Left uterine horn; RUH, right uterine horn.

**Figure 4:**
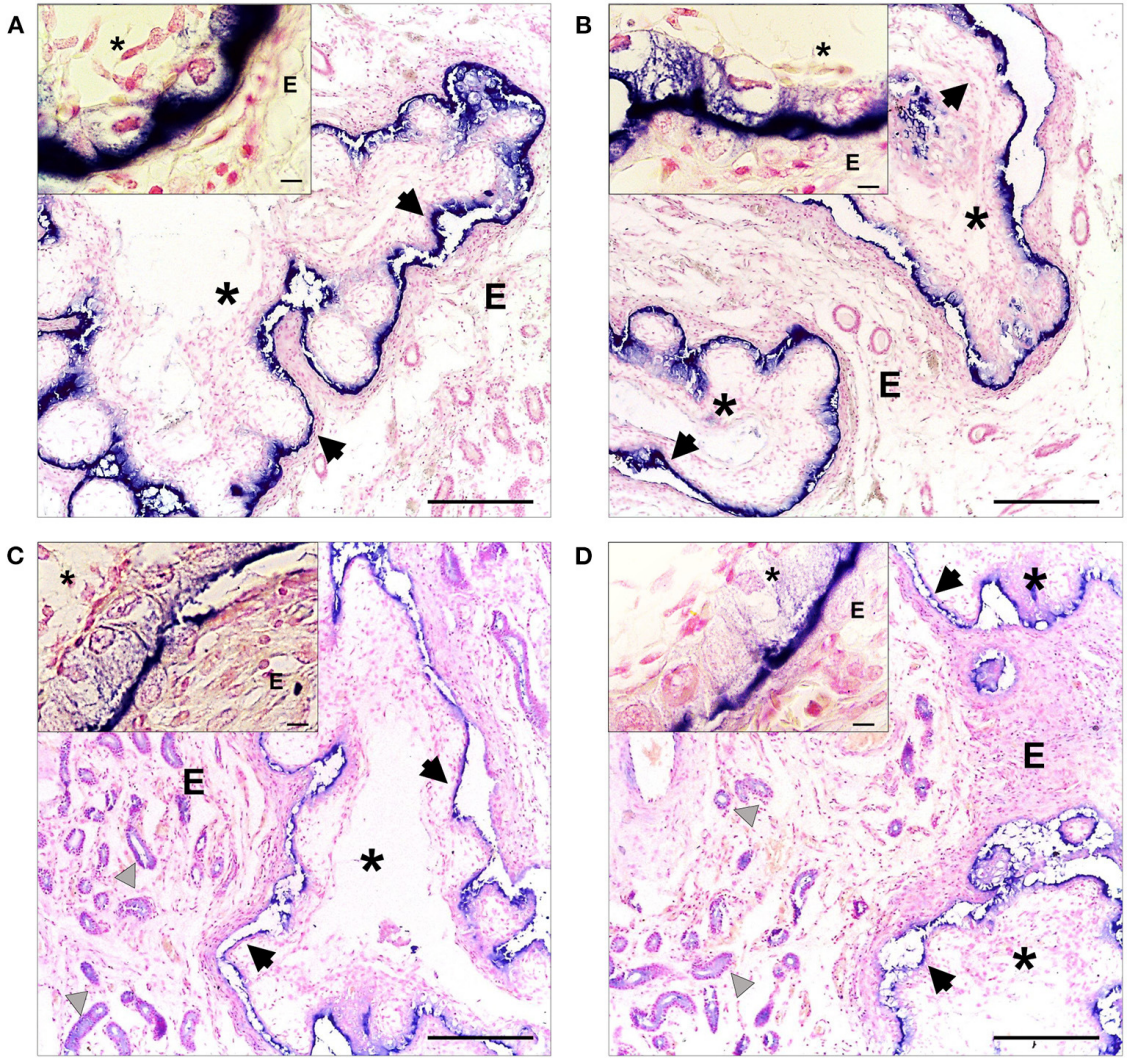
Immunohistochemical localization of β-NGF **(A,B)** and TrKA **(C,D)** in the endometrial mucosa of 30 days-pregnant alpacas, positive labeling gives a blue-violaceous coloration. 100-fold magnification (bar = 100 μm) of left uterine horn **(A,C)** and right uterine horn **(B,D)**. In addition, inset shows a magnification of the extraembryonic membranes (bar = 10 μm). E: endometrium, asterisks: extraembryonic membranes, black arrows indicate the positive immunolabeling on the extraembryonic membranes, gray triangles indicate positive TrKA immunostaining in glandular epithelium and vessels.

In 30 days-pregnant alpacas, a strong immunosignal was observed in the extraembryonic membranes of the conceptus for β-NGF (Figures 4A,B) and TrKA (Figures 4C,D).

### Relative Abundance of *VEGFA* in Alpaca Endometrium

**Figure 5:**
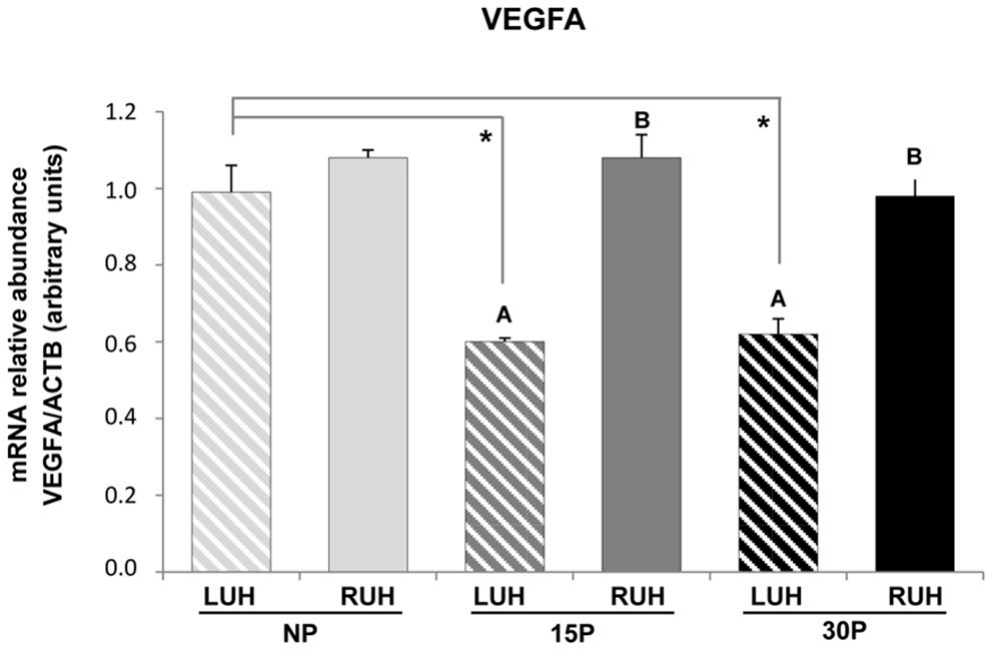
Relative abundance of *VEGFA* mRNA in the endometrium of non-pregnant (NP), 15 days-pregnant (15P), and 30 days-pregnant (30P) alpacas by RT-PCR. Histograms illustrate mean values ± SE. Different uppercase letters **(A,B)** indicate significant differences between LUH (left uterine horn) and RUH (right uterine horn) within NP, 15P, and 30P status (*p* < *0.05*). Asterisks indicate significant differences (*p < *0.05*) between the physiological status.

*VEGFA* transcripts were present in the endometrium of uterine horns from non-pregnant and pregnant alpacas (Figure 5). Differences between left and right uterine horns were observed only in pregnant alpacas (15 and 30P), in all cases, the RUH showed a higher relative abundance than the LUH (*p* ≤ 0.01).

When the different physiological status were compared, only the LUH of non-pregnant females displayed a higher relative abundance of *VEGFA* than that of LUH of pregnant alpacas. The RUH presented similar relative abundance in all the experimental groups. No statistically significant association was observed between β-NGF expression and *VEGFA* relative abundance (Table 4).

**Table 4:** Pearson correlation coefficient between β-NGF and VEGFA, blood vessel area and number for left and right uterine horns.

		**VEGFA mRNA relative abundance**		**Area of blood vessels**		**Number of blood vessels**	
		**LUH**	**RUH**	**LUH**	**RUH**	**LUH**	**RUH**
β-NGF mRNA relative abundance	LUH	−0.492	−0.201	0.446	0.475	0.092	−0.453
	RUH	−0.576	−0.290	0.816^**^	0.853^**^	−0.168	−0.430
β-NGF immunosignal density	LUH	−0.220	0.373	−0.633^*^	−0.632^*^	0.612^*^	0.578^*^
	RUH	−0.460	0.356	−0.411	−0.412	0.546	0.431

*LUH, Left uterine horn; RUH, right uterine horn*.

** *p < 0.01 (bilateral)*.

* *p < 0.05 (bilateral)*.

### Vessels Abundance and Distribution

**Figure 6:**
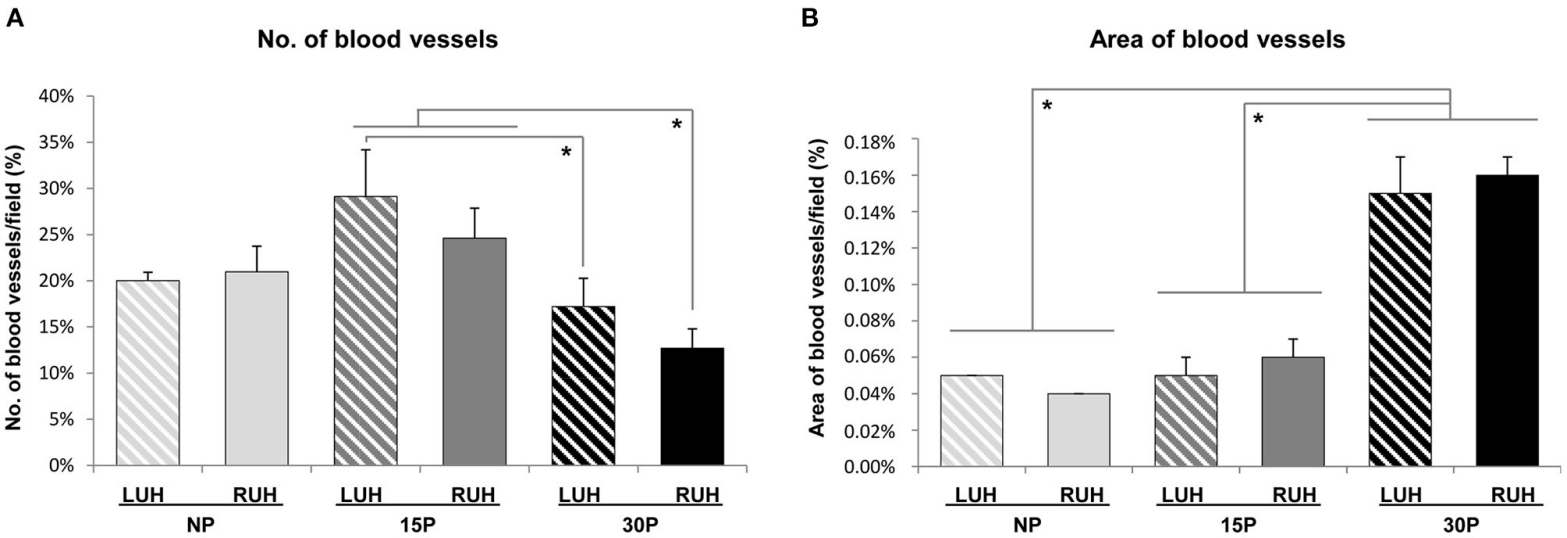
Effect of pregnancy status and stage of pregnancy on the blood vessel number and area in the endometrium of alpacas. **(A)** Number of blood vessels in LUH (left uterine horn) and RUH (right uterine horn) of non-pregnant (NP), 15 days-pregnant (15P), and 30 days-pregnant (30P) alpacas. **(B)** Percentage of the endometrial area occupied by blood vessels in LUH and RUH. Histograms illustrate mean values ± SE. Asterisks indicate significant differences (*p < *0.05*).

The number of blood vessels was similar for LUH and RUH in the 3 groups studied. The endometrium of 30 days-pregnant alpacas showed fewer vessels than 15 day-pregnant females. 30P-RUH was significantly different from both 15P-RUH and 15P-LUH. Whereas, 30P-LUH presented a smaller number of vessels than 15-LUH (Figure 6A).

The area occupied by the vessels was similar between non-pregnant and 15 days-pregnant females. While 30 days-pregnant animals presented significantly higher values of the vascularized area compared to non-pregnant and 15 day-pregnant alpacas. No differences between left and right uterine horns were detected (Figure 6B).

LUH displayed a positive association between β-NGF immunolabelling and the number of vessels (*p* = 0.034); however, the correlation between β-NGF immunolabelling and the vascularized area of the endometrium was negative (*p* = 0.027). Regarding RUH, no statistically significant association was observed between β-NGF immunosignal and the number or area of vessels (Table 4).

## Discussion

The current study reveals for the first time the gene expression patterns and the protein localization of β-NGF and TrKA in the alpaca uterus during the peri-implantation period along with *VEGFA* gene expression patterns and the features of endometrium vasculature. These results support the implication of the β-NGF system during the early pregnancy period of South American camelids.

β-NGF and TrKA presence has been reported in the uterus of spontaneous and reflex ovulatory species, as well as in species with different placentation, such as humans, rodents, and rabbits with hemochorial placentation, goats and bovines with synepitheliochorial placentation, and pigs and horses with epitheliochorial placentation (17, 20, 36, 37). In all the species assayed, β-NGF immunoreactivity was detected in the luminal epithelium, glandular epithelium, and vascular tissue, this latter especially in pig and horse. The uterine expression of TrKA mirrored that of its ligand, it is mainly localized in the luminal, glandular epithelium, and vessels of the above-mentioned species (17, 20, 36, 37). Coincidently, the same distribution was observed for β-NGF system in alpaca oviducts (38), and uterine horns. In camelids, the establishment of pregnancies occurs almost exclusively in the LUH; at 15 days of pregnancy elongated embryos, that reach a length of 21 cm, are free within the uterine lumen until day 22–26 of gestation when the trophoblast becomes apposed to the epithelial surface of the uterus (6, 7). Striking differences were determined in alpacas for β-NGF and TrKA immunolabeling among the different physiological status analyzed. The signal was remarkably strong in the uterus of 15 days-pregnant females when the embryo elongates and the endometrium prepares for implantation. In contrast, it was faint in non-pregnant and 30 days-pregnant alpacas. In squirrel (*Citellus dauricus Brandt*), β-NGF and TrKA proteins reached the highest immunoreactivity at the time of early pregnancy (15). In coincidence, Lobos et al. (18) reported higher amounts of mature β-NGF in the uterus from early pregnant rats compared to non-pregnant, middle, and late pregnant females. Interestingly, although β-NGF protein was almost imperceptible in the uterus of 30 days-pregnant alpacas, a strong signal was ascertained in the extraembryonic membranes, as well as for TrKA signal. According to this, in humans, β-NGF was found in the cyto and syncytial trophoblast, chorionic mesodermic cells, and in decidua during the first trimester of gestation (39). Moreover, Coassin et al. (40) also reported the presence of β-NGF and TrKA in human amniotic membranes. In alpacas, even though no differences were observed in the β-NGF immunohistochemical patterns between the LUH and the RUH during pregnancy, TrKA immunolabeling was stronger in the LUH of non-pregnant and 15 days-pregnant alpacas. Maranesi et al. (17), demonstrated that treatment with TrKA inhibitor reduced the NGF-induced pathway in rabbit uterus. In addition, gene expression studies of matrix metalloproteinases (MMPs) during early pregnancy in alpacas (*Vicugna pacos*), demonstrated higher mRNA levels of MMP-2, which play an important role during embryo implantation in the uterine horn, in the LUH compared to the RUH of non-pregnant and 15 days-pregnant alpacas (41). Interestingly, MMP-2 activates pro-NGF into NGF (42). All these data would suggest that the NGF pathway is more active in the luminal epithelium of the left uterine horn, especially in the pre-implantation period, when the immunolabelling of β-NGF and TrKA was increased.

It has been suggested that β-NGF function in the uterus and placenta during pregnancy is to ensure adequate maternal immunomodulatory and developmental processes at the fetal-maternal interface. A well-balanced level of β-NGF is required for a successful pregnancy outcome as both insufficient or elevated levels of the factor may provoke fetal rejection (2, 21, 43). In alpacas the β-NGF system was not only detected in embryo membranes when the implantation has already occurred but also in the pre-implantation endometrium, during embryo elongation, suggesting a role during this period.

Intriguingly, in alpaca endometrium β-NGF and TrKA mRNA and protein expression were dissimilar, analogous observations were reported by Sari et al. (38) when analyzing gene and protein expression pattern in the llama oviduct. Many authors have postulated that regulation of β-NGF production does not occur solely at the level of transcription and that post-transcriptional mechanisms operate as well. Even more, there is a diverse post-transcriptional regulation between different cell types (44–46). For example, in rat uterus, alterations in NGF isoforms during pregnancy, accumulation of proNGF, and decreased ratios of mature β-NGF to proNGF were reported (18).

A rise in endometrial vascularization during pregnancy has been described in other mammals. Mares that were pregnant exhibited a greater area of the endometrium occupied by blood vessels compared with non-pregnant females and mares at 21 days of pregnancy exhibited the greatest area of endometrium occupied by blood vessels (47). Something similar occurs in cows, in which the number of blood vessels in the endometrium increased from day 15 to 18 of gestation, and pregnant cows had more blood vessels than non-pregnant cows on day 18 (48, 49). Likewise, in alpacas, a larger area of the endometrium was occupied by blood vessels at 30 days of gestation. It is known that intrauterine administration of β-NGF induces an increase of endometrial vascularization in both uterine horns (13) and it has been demonstrated that β-NGF acts as a pro-angiogenic factor that increases the expression of VEGFA in cultures of llama granulosa cells (29). In 15 days-pregnant alpacas, the increased β-NGF protein intensity was in correspondence with the elevated number of vessels in the endometrium. At 15 days of pregnancy, embryos occupied the entire uterine cavity and on day 45 the entire extent of the trophoblast was closely attached to the left and right uterine epithelium (6, 7). This could explain the vascular similarities between both uterine horns; nevertheless, a positive correspondence between β-NGF and the number of vessels was only observed in the left uterine horn.

The VEGF family is known to regulate vascular functions such as angiogenesis, vasculogenesis, vascular permeability, and lymphangiogenesis; and is composed of several subtypes including VEGFA, VEGFB, VEGFC, VEGFD, and placental growth factor (50). *VEGFA* mRNA can be alternatively spliced into five iso-forms representing proteins of 121, 145, 165, 189, and 206 amino acids (51). In alpacas*, VEGFA* mRNA was present in the uterus of pregnant and non-pregnant females, showing a higher expression in the endometrium of non-pregnant animals and differences between horns in pregnant females. In bovines, gene expression of *VEGFA* transcript variant 1 was higher in non-pregnant females (18th day of estrous cycle) compared with pregnant ones (18th day of pregnancy). Even more, *VEGFA* levels did not show changes during early pregnancy or during the peri-implantation period (49). Hayashi et al. (48) speculated that the lower peripheral estradiol concentrations in pregnant heifers explain these results. In addition to this, Johnson et al. (52) determined that the expression of *VEGFA* in the endometrium of ovariectomized sheep was stimulated by estradiol. Moreover, estradiol receptor, mRNA expression was lower in the ipsilateral uterine horn of non-pregnant sheep, cows and horses, and in pregnant mares (day 13), indicating changes in the sensitivity of the endometrium to estradiol (53). These findings could also explain the differences of *VEGFA* expression between uterine horns and physiological status in alpacas. In mares and pigs, *VEGFA* was upregulated during early pregnancy. In pigs *VEGFA* isoform 188 aa was specifically assayed (46); while in horses, the *VEGFA* transcript variant X1 was amplified (54). In alpacas, primers amplified *VEGFA* transcript variant X1(186 aa), X2 (180 aa), and X3 (162 aa), a fact that could also explain the differences between alpacas and other livestock species. Although the *VEGFA* gene expression pattern did not show drastic changes between pregnancy periods and was even decreased compared to non-pregnant alpacas, other members of the VEGF system could be involved in the angiogenesis changes observed in alpacas. Hayashi et al. (48), reported an increase of *VEGFB* on day 15 compared to days 18 and 27 of pregnancy, suggesting the possible involvement of VEGFB in endometrial receptivity for successful implantation at earlier stages in Japanese Black cows.

In summary, the endometrium expresses β-NGF, TrKA, and VEGFA in pregnant and non-pregnant alpacas. β-NGF differential protein pattern in alpaca endometrium during the peri-implantation period of pregnancy implies a precise regulation, which suggests a possible role in embryo development and implantation. The proangiogenic function of β-NGF could be responsible for the increase of endometrial vasculature, thereby supporting embryo implantation and survival. Nevertheless, further investigation on the β-NGF and VEGF signaling pathway is necessary to fully understand the differential implantation in South American camelids.

## Data Availability Statement

The raw data supporting the conclusions of this article will be made available by the authors, without undue reservation.

## Ethics Statement

The animal study was reviewed and approved by Committee of Animal Ethics and Welfare (Comité de Ética y Bienestar Animal (CEBA) from the School of Veterinary Medicine of the UNMSM. Written informed consent for participation was not obtained from the owners because the animals were destined for meat production and they were sent to a slaughterhouse, where samples were collected following the regulations of the abattoir.

## Author Contributions

DB and MA analyzed the data and wrote the manuscript. MA, LS, and DB performed the laboratory analysis and participated in the acquisition of the data. MA and SA designed the study. SA and MR discussed and revised the manuscript. All authors read and approved the manuscript for publication.

## Conflict of Interest

The authors declare that the research was conducted in the absence of any commercial or financial relationships that could be construed as a potential conflict of interest.
